# The Effectiveness of National Early Warning Score, Quick Sequential Organ Failure Assessment, Charlson Comorbidity Index, and Elixhauser Comorbidity Index Scores in Predicting Mortality Due to COVID-19 in Elderly Patients

**DOI:** 10.7759/cureus.23012

**Published:** 2022-03-09

**Authors:** Canan Akman, Okan Bardakçı, Murat Daş, Gökhan Akdur, Okhan Akdur

**Affiliations:** 1 Emergency Department, Canakkale Onsekiz Mart University Faculty of Medicine, Canakkale, TUR

**Keywords:** mortality, emergency medical service, early warning score, comorbidity models, age

## Abstract

Introduction: As the mortality rate in coronavirus disease 2019 (COVID-19) patients older than 65 years is considerable, evaluation of in-hospital mortality is crucial. This study aimed to evaluate in-hospital mortality in COVID-19 patients older than 65 years using the National Early Warning Score (NEWS), Quick Sequential Organ Failure Assessment (q-SOFA), Charlson Comorbidity Index (CCI), and Elixhauser Comorbidity Index (ECI).

Methods: This retrospective study included data from 480 patients with confirmed COVID-19 and age over 65 years who were evaluated in a university emergency department in Turkey. Data from eligible but deceased COVID-19 patients was also included. NEWS, q-SOFA, CCI, and ECI scores were retrospectively calculated. All clinical data was accessed from the information management system of the hospital, retrieved, and analyzed.

Results: In-hospital mortality was seen in 169 patients (169/480). Low oxygen saturation, high C-reactive protein (CRP) and urea levels, and high q-SOFA and ECI scores helped us identify mortality in high-risk patients. A statistically significant difference was found in mortality estimation between q-SOFA and ECI (p <0.001), respectively.

Conclusion: Q-SOFA and ECI can be used both easily and practically in the early diagnosis of in-hospital mortality in COVID-19 positive patients over 65 years of age admitted to the emergency department. Low oxygen saturation, high CRP and urea levels, and high q-SOFA and ECI scores are helpful in identifying high-risk patients.

## Introduction

Severe acute respiratory syndrome coronavirus 2 (SARS-CoV-2), coronavirus disease 2019 (COVID-19), which initially emerged in Wuhan, China, and then later has spread all over the world and eventually declared as a pandemic [1]. The progression of COVID-19 can be critical, especially in patients aged over 65 years [2]. While studies have revealed that those who were admitted to the intensive care unit were older, the average age of those who died has been reported to be 64.6 years, and age >60 has been identified as a risk factor that is associated with poor prognosis and in-hospital mortality [3,4,5], with the highest mortality rate in elderly patients [6]. Thus, early estimation of in-hospital mortality in patients admitted to the emergency department who may need intensive care would be beneficial and can also contribute to the planning of health services within the hospital under pandemic conditions. The Charlson Comorbidity Index (CCI) and the Elixhauser Comorbidity Index (ECI) [7,8,9] are the most commonly used tools for evaluating risk and in-hospital mortality. The CCI is simple, easy, and accurate, and can predict long-term prognosis and survival by estimating the risk of mortality from a comorbid disease [10]. The ECI estimates in-hospital mortality in the presence of 30 comorbid conditions [5], and it has been suggested that the National Early Warning Score (NEWS) and the Quick Sequential Organ Failure Assessment (q-SOFA) can be used to predict mortality [11,12]. The number of COVID-19 cases and mortality has recently increased all over the world, and it has been observed that severe cases have high mortality rates [5]. Thus, we aimed to compare the ability of NEWS, q-SOFA, CCI, and ECI in predicting mortality in patients aged over 65 years, who presented to the emergency department, were diagnosed with COVID-19, and required intensive care.

## Materials and methods

Study design

This retrospective cross-sectional study was initiated after obtaining approval from the Canakkale Onsekiz Mart University Clinical Research Ethics Committee (date of approval: 05.02.2021, No:2021-01). All patients aged over 65 years who presented to the emergency department of the university hospital with a diagnosis of COVID-19 between July 2020 and May 2021 were included in the study. Demographic characteristics of the patients and clinical findings, vital signs, serum biochemistry, hemogram, blood gas values, and emergency service and intensive care results were analyzed. Both NEWS and q-SOFA scores were calculated for each patient to assess COVID-19 severity, and CCI and ECI were used to predict in-hospital mortality.

Patients

Patient information was obtained from the hospital information management system. COVID-19 patients aged over 65 years with a positive polymerase chain reaction test were included in the study, whereas those younger than 65 years, trauma patients, those not diagnosed with COVID-19, or those whose data could not be accessed or were missing from the hospital information management system were excluded from the study.

Laboratory analysis

Serum biochemistry, hemogram, and blood gas values of the patients were estimated in the biochemistry laboratory. Serum biochemistry was analyzed using the colorimetric method (501 module of the Roche Cobas 6000 device (Roche Diagnostics, Poland)), hemogram was derived with the electrical impedance method (Beckman Coulter DXH 800 device, USA), and blood gas values were quantified using the ion-selective electrode method in a ABL800 device (Radiometer, Denmark).

Statistics

Numerical variables are expressed as the median and interquartile range (IQR) and categorical variables as numbers and percentages. Categorical variables were evaluated using Pearson’s chi-square test or Fisher’s exact test. The Shapiro-Wilk test was used to assess the normality assumption for the continuous variables. Differences between two groups for non-normally distributed continuous variables were evaluated by the Mann-Whitney U test. Logistic regression was used to predict mortality and relationship, which are indicated by univariate and multivariate odds ratios at 95% confidence intervals. A base model was created by adding age and gender to statistically significant variables, namely, urea, C-reactive protein (CRP), and oxygen saturation, during multivariable analysis. Receiver operating characteristic (ROC) analysis was used to test the ability of this base model to predict mortality and that of the models created by adding q-SOFA or ECI to the base model. The Delong test was used for pairwise comparison of the area under the curve (AUC) in the receiver operating characteristic (ROC) analysis of the base model, which we constructed by using age, gender, urea, CRP and oxygen saturation for mortality estimation [13]. All statistical analyses were performed on SPSS 26.0 for Windows (IBM Corp, Armonk, NY, USA). All p-values of <0.05 were considered statistically significant.

## Results

We included data from 480 eligible patients. In-hospital mortality occurred in 169 (169/480) of these patients, their median age was 77.0 years (IQR: 71.0-83.0), and (33.7%) were female (p<0.001). Comparison of vital parameters at admission revealed a statistically significant difference in oxygen saturation, respiratory rate, systolic blood pressure (SBP), and diastolic blood pressure (DBP) between those who died and survived (p<0.001, p<0.001, p=0.008, and p=0.040, respectively). Differences in laboratory parameters were seen in lymphopenia (p<0.001) with significantly higher levels of lactate, base deficit, CRP, aspartate aminotransferase (AST), urea, creatinine, and D-dimer levels seen in patients who died. The survival rate of hospitalized patients was found to be 29.8% among patients initially admitted to the emergency service and 24.7% in those who were initially admitted to the intensive care unit (p<0.001). Further, those who died had higher NEWS, q-SOFA, CCI, and ECI scores (p=0.001 for NEWS, p<0.001, for all others) (Table 1).

**Table 1:** Vital parameters, laboratory parameters, evaluation patients, and scores SBP: systolic blood pressure; DBP: diastolic blood pressure; CRP: c-reactive protein; ALT: alanine aminotransferase; AST: aspartate aminotransferase; LOS in ICU: length of stay in ıntensive care unit; NEWS: national early warning score; Q-SOFA: quick sequential organ failure assessment; ECI: Elixhauser comorbidity ındex; CCI: Charlson comorbidity ındex

	All patients n = 480 (100%)	Survived n = 311 (64.8%)	Deceased n = 169 (35.2%)	p-value
	Median (IQR/n(%))	Median (IQR)/n(%))	Median (IQR/n(%))	
Age (year)	74.0 (68.0-81.0)	72.0 (67.0-80.0)	77.0 (71.0-83.0)	<0.001
Gender				
Female	207 (43.1)	138 (66.7)	69 (33.7)	0.454
Male	273 (56.9)	173 (63.4)	100 (36.6)	
Vital signs at triage				
Saturation (%)	94.0 (90.0-97.0)	98.0 (95.0-98.0)	92.0 (85.0-96.0)	<0.001
Heart rate (beat/min)	87.0 (78.0-99.8)	87.0 (78.0-98.0)	104.5 (88.0-125.0)	0.085
Respiratory (rate/min)	22.0 (18.0-24.0)	20.0 (18.0-22.0)	22.0 (20.0-26.0)	<0.001
SBP (mmHg)	132.0 (115.3-148.0)	133.0 (119.0-149.0)	130.0 (109.5-146.5)	0.008
DBP (mmHg)	78.0 (67.0-87.0)	78.0 (70.0-87.0)	76.0 (64.0-85.0)	0.040
Laboratory parameters				
Lymphocyte (×10^3^/µL)	1.0 (0.6-1.6)	1.1 (0.7-1.7)	0.8 (0.5-1.3)	<0.001
Lactate (mmol/L)	1.6 (1.2-2.3)	1.5 (1.1-2.2)	1.8 (1.4-2.7)	<0.001
Base deficit (mmol/L)	0.05 (-2.6 to -2.4)	0.6 (-1.7 to -2.8)	-1.2 (-4.9 to -1.6)	<0.001
CRP (mg/dL)	8.1 (3.53-15.7)	6.18 (2.6-13.5)	11.5 (6.5-20.34)	<0.001
ALT (U/L)	18.9 (12.0-28.9)	18.9 (11.8-26.9)	19.0 (12.7-34.1)	0.115
AST (U/L)	28.1 (20.0-41.7)	26.9 (19.4-39.1)	30.1 (21.1-50.9)	0.005
Urea (mg/dL)	50.1 (35.6-75.9)	44.1 (33.2-67.4)	66.3 (43.7-103.5)	<0.001
Creatinine (mg/dL)	1.12 (0.84-1.63)	0.98 (0.78-1.38)	1.30 (0.94-1.94)	<0.001
D-dimer (ng/mL)	413.5 (198.3-852.3)	374.0 (185.0-668.0)	535.0 (254.0-1306.0)	<0.001
CT (involvement)	468 (100%)	302 (64.5%)	166 (35.5%)	0.453
Disposition				
Discharge	37 (7.7%)	37 (100%)	0 (0.0%)	<0.001
Regular ward	362 (75.4%)	254 (70.2%)	108 (29.8%)	
ICU	81 (16.9%)	20 (24.7%)	61 (75.3%)	
LOS in ICU	0.0 (0.0-6.0)	0.0 (0.0-0.0)	7.0 (1.0-14.0)	<0.001
Illness acuity assessment tools				
NEWS	8.0 (6.0-10.0)	7.0 (6.0-10.0)	8.0 (6.0-11.0)	0.001
q-SOFA	1.0 (0.0-1.0)	1.0 (0.0-1.0)	1.0 (1.0-2.0)	<0.001
ECI	5.0 (0.0-10.0)	3.0 (0.0-8.0)	8.0 (5.0-12.0)	<0.001
CCI	4.0 (3.0-5.0)	4.0 (3.0-5.0)	5.0 (4.0-6.0)	<0.001

These parameters were subjected to univariate and multivariate analyses to identify factors associated with survival and risk of mortality. Multivariate analysis revealed that while a one-unit decrease in oxygen saturation increased mortality risk by 8.9%, a single-unit increase in CRP raised the risk by 1.043 (1.015-1.072) times. One-unit increase in CRP was 1.043 (1.015-1.072) times, one-unit increase in urea was 1.008 (1.002-1.014) times, and q-SOFA scores were higher. It was determined that an increase of one point increased the risk of mortality by 3.934 (2.681-5.771) times, and an increase of one point in ECI increased the risk of mortality 1.111 (1.063-1.161) times (Table 2).

**Table 2:** Variables affecting mortality risk SBP: systolic blood pressure; DBP: diastolic blood pressure; CRP: c-reactive protein; ALT: alanine aminotransferase; AST: aspartate aminotransferase; LOS in ICU: length of stay in ıntensive care unit; NEWS: national early warning score; Q-SOFA: quick sequential organ failure assessment; ECI: Elixhauser comorbidity ındex; CCI: Charlson comorbidity ındex

Mortality				
	Univariate		Multivariate	
	Odds ratio (95% CI)	p-value	Odds ratio (95% CI)	p-value
Demographic				
Age	1.049 (1.025-1.074)	<0.001		
Gender/Female (Ref)	1.156 (0.791-1.690)	0.454		
Vital sign at the triage				
SBP	0.987 (0.979-0.995)	0.001		
DBP	0.986 (0.974-0.998)	0.024		
Heart rate	1.013 (1.003-1.023)	0.008		
Respiratory rate	1.138 (1.080-1.200)	<0.001		
Oxygen saturation (%)	0.917 (0.891-0.945)	<0.001	0.911 (0.870-0.954)	<0.001
Laboratory parameters				
Lymphocyte	1.018 (0.978-1.060)	0.377		
Lactate	1.247 (1.084-1.436)	0.002		
Base deficit	0.909 (0.874-0.946)	<0.001		
CRP	1.060 (1.038-1.083)	<0.001	1.043 (1.015-1.072)	0.002
AST	1.005 (1.000-1.010)	0.039		
ALT	1.005 (1.000-1.010)	0.063		
Urea	1.016 (1.011-1.021)	<0.001	1.008 (1.002-1.014)	0.006
Creatinine	1.542 (1.281-1.857)	<0.001		
D-dimer	1.000 (1.000-1.000)	0.293		
LOS in ICU	1.154 (1.114-1.196)	< 0.001		
Illness acuity assessment tools				
NEWS	1.131 (1.054-1.215)	0.001		
q-SOFA	4.276 (3.091-5.914)	<0.001	3.934 (2.681-5.771)	<0.001
ECI	1.145 (1.104-1.188)	<0.001	1.111 (1.063-1.161)	<0.001
CCI	1.339 (1.197-1.499)	<0.001		

ROC analysis of the base model constructed using age, gender, urea, CRP, and oxygen saturation for mortality estimation had an AUC value of 0.757, and this value increased to 0.809 upon the addition of q-SOFA to the base model, and to 0.793 after adding ECI. Notably, a statistically significant difference was observed in the prediction of mortality between these two scores (p=0.002, p=0.009, respectively) (Table 3).

**Table 3:** Comparison of discrimination accuracy between ECI and Q-SOFA scores ECI: Elixhauser comorbidity ındex; q-SOFA: quick sequential organ failure assessment; CRP: c-reactive protein; DBA: difference between area; SE: standard error; ROC: receiver operating characteristic

Base model = age, gender, urea, CRP, saturation	Area under the ROC curve (95% CI)	Area under the ROC curve (95% CI)	Pairwise analysis					
			DBA	SE	95% CI		Z statistics	p-value
	Without q-SOFA score	With q-SOFA score			Lower	Upper		
	0.757 (0.712-0.802)	0.809 (0.768-0.851)	-0.052	0.209	-0.086	-0.019	-3.062	0.002
	Area under the ROC curve (95% CI)	Area under the ROC curve (95% CI)	Pairwise analysis					
	Without ECI score	With ECI score						
	0.757 (0.712-0.802)	0.793 (0.753-0.834)	-0.036	0.207	-0.063	-0.009	-2. 604	0.009

## Discussion

Among the elderly, COVID-19 progression is characterized by a more serious clinical profile, specifically, intensive care admissions and length of hospital stay are prolonged, apart from higher mortality. Previous studies have established a link between mortality rate and age, advanced age is labeled as a poor prognostic indicator [5,14,15]. A retrospective study by Berenguer et al. among 4035 patients with an average age of 70 years showed that mortality increased with age and that it was 85.6% in patients aged over 65 years [16]. We report a 77% mortality rate and our results correlate with reported values. Two studies, one each by Chen et al. and Mendes et al., state that male gender is a risk factor for mortality due to COVID-19 [17,18], and the mortality rate was higher in males, aged over 65 years, and this pattern was consistent in all age groups.

An analysis of the vital signs of the patients who died revealed that the average respiratory rate was 22/min, oxygen saturation was 92%, SBP was 130 mmHg, and DBP was 76 mmHg. In the study by Chen et al. among 113 patients with an average age of 68 years who died from COVID-19, the average respiratory rate was <24/min, oxygen saturation was ≤93%, and SBP was 137 mmHg [19]. Further, in a cohort of 334 patients whose average age was 66 years, the respiratory rate was 20/min, oxygen saturation was 89%, and SBP and DBP were 128 mmHg and 73 mmHg, respectively [20]. Our results concur with those reported previously and we also describe significant lymphopenia, along with higher levels of AST, serum urea, creatinine, D-dimer concentration, CRP, and base deficit among deceased patients [19,21,22,23]. In a report organized by the Center for Disease Control and Prevention in the United States (CDC USA), it was stated that 80% of the elderly patients admitted to the intensive care unit died [24]. We report a similar mortality rate of 75.3% and these figures are remarkably high.

The NEWS score can accurately predict in-hospital mortality and intensive care hospitalization in emergency services [25,26]. Further, Zhou et al. analyzed 54 deaths among 191 patients and report that the q-SOFA score is associated with mortality [4]. Kuswardhani et al. have shown that mortality and severity of COVID-19 disease were associated with higher CCI scores [10], and Qeadan et al. state that the risk of mortality from COVID-19 was greater in elderly patients with a high ECI score [27]. Consistently, we also show that NEWS, q-SOFA, CCI, and ECI scores were higher in deceased patients.

Low oxygen saturation in COVID-19 patients is independently associated with in-hospital high mortality [28]. CRP, it increases in mortality due to hyperinflammation, regardless of age or gender. Therefore, it can be used safely to predict in-hospital mortality [29]. In severe and fatal cases, an increase in kidney function parameters (urea, creatinine) results in multiorgan failure [30]. Sepsis scores are useful in estimating in-hospital mortality and it is important to identify patients at risk, quickly and simply, but in intensive care, the SOFA score is more suitable [31,32]. The use of q-SOFA is advantageous in the emergency room because it does not require laboratory test data and can predict poor prognosis. Importantly, studies reveal that, although the sensitivity of q-SOFA is low, its specificity is high in general [31], and Zhou et al. have shown that high q-SOFA was associated with high mortality [33,34,35], and both CCI and ECI can provide mortality prognosis based on the presence of comorbidities in the patient population. While available literature on acute and chronic conditions indicates that the ECI is statistically superior in predicting mortality [9], we found that q-SOFA more accurately predicted mortality than the ECI. Elixhauser et al. have used comorbidities to evaluate in-hospital mortality, length of hospital stay, and estimated medical expenses [8], and a systematic analysis has indicated that the ECI can adequately predict in-hospital mortality [36]. In our study, in-hospital mortality increased with low oxygen saturation, CRP raised and serum urea levels increased, higher-level q-SOFA and ECI scores were seen in deceased patients.

The fact that it is single-centered is the limitation of our study.

## Conclusions

In the chaotic environment of the emergency department, early diagnosis and determination of mortality due to COVID-19 in patients aged over 65 years are important. While the ECI is widely used to predict mortality in COVID patients with comorbidities, we believe that the use of q-SOFA, along with ECI in the emergency department will facilitate as simple and practical tools in early detection of in-hospital mortality.
